# Diagnosing schizophrenia with routine blood tests: a comparative analysis of machine learning algorithms

**DOI:** 10.3389/fpsyt.2025.1630922

**Published:** 2025-08-19

**Authors:** Yavuz Selim Ogur, Ayse Erdogan Kaya, Nur Banu Ogur, Beyza Erdogan Akturk

**Affiliations:** ^1^ Department of Psychiatry, Serdivan State Hospital, Sakarya, Türkiye; ^2^ Department of Psychiatry, Faculty of Medicine, Hitit University Çorum Erol Olçok Training and Research Hospital, Çorum, Türkiye; ^3^ Department of Software Engineering, Faculty of Computer and Information Sciences, Sakarya University, Sakarya, Türkiye; ^4^ Department of Psychiatry, Tarsus State Hospital, Mersin, Türkiye

**Keywords:** schizophrenia, machine learning, biomarkers, grey wolf optimization (GWO), blood parameters

## Abstract

**Introduction:**

Schizophrenia is a severe mental disorder affecting approximately 1% of the general population, diagnosed primarily using clinical criteria. Due to the lack of objective diagnostic methods and reliable biomarkers, accurate diagnosis and effective treatment remain challenging. Peripheral blood biomarkers have recently attracted attention, and machine learning methods offer promising analytical capabilities to enhance diagnostic accuracy.

**Methods:**

This retrospective, case-control study included 203 schizophrenia patients treated over a five-year period at a tertiary hospital and 192 age- and sex-matched healthy controls. Demographic data and routine hematological and biochemical parameters were extracted from medical records. Variables missing more than 85% of data were excluded; remaining missing values were imputed after train-test splitting to avoid data leakage. Optimal biomarker subsets were selected using Grey Wolf Optimization (GWO). Random Forest (RF), XGBoost, Support Vector Machine (SVM), K-Nearest Neighbor (KNN), and Logistic Regression (LR) models were trained and evaluated via stratified 10-fold cross-validation.

**Results:**

Groups were homogeneous in terms of age and sex. Before GWO optimization, XGBoost (95.55%) and Random Forest (94.63%) yielded the highest accuracies. Following optimization, Random Forest accuracy improved (94.95%) with a recall of 96.25%, while XGBoost reached the highest accuracy (95.90%) and strong specificity (95.54%). Post-optimization, Area Under the Curve (AUC) values were highest for XGBoost (0.96) and Random Forest (0.95), indicating strong diagnostic performance. Total protein, glucose, iron, creatine kinase, total bilirubin, uric acid, calcium, and sodium were key biomarkers distinguishing schizophrenia. Interestingly, glucose levels were significantly lower in schizophrenia patients compared to controls, contrary to typical findings. Differences in triglycerides, liver enzymes, sodium, and potassium lacked clear clinical significance.

**Discussion:**

The machine learning models developed provided diagnostic accuracy comparable to studies utilizing more expensive biomarkers, highlighting potential clinical and economic advantages. External validation is recommended to further confirm the generalizability and clinical utility of these findings.

## 1 Introduction

Schizophrenia is a severe and complex mental disorder characterized by a high prevalence (approximately 1% lifetime prevalence) and significant functional impairment, imposing a substantial burden on both individuals and society ​ (1). Currently, there are no definitive biomarkers, neuroimaging findings, or laboratory tests that can objectively confirm the diagnosis of psychiatric disorders such as schizophrenia. Diagnosis relies entirely on clinical assessment based on standardized criteria systems like DSM-5 or ICD-10 (2–4). This diagnostic approach presents significant challenges due to the substantial symptom overlap between schizophrenia and other psychiatric disorders (5, 6). Indeed, the etiopathogenesis of schizophrenia remains incompletely understood, and consequently no effective, specific, and objective biomarker has yet been identified. This critical gap represents a global scientific challenge that significantly hinders the development of precise diagnostic tools and targeted therapies (1, 7).​

Biomarker-based approaches have become a major research focus in recent years to objectify schizophrenia diagnosis and reduce reliance on clinicians’ subjective assessments. Various biomarker candidates have been investigated, including genetic susceptibility markers, metabolic and endocrine indicators, neuroimaging findings, and electrophysiological characteristics ​ (1). Particularly in the domain of peripheral blood biomarkers, novel findings are shedding light on the biological underpinnings of schizophrenia. While the disorder was historically attributed to dysregulation of dopaminergic, glutamatergic, or serotonergic neurotransmission, emerging evidence from the past decade strongly implicates immune system abnormalities in schizophrenia pathogenesis (5, 8, 9).​. In this context, potential blood-based biomarkers that may reflect disease pathogenesis are being intensively investigated. For instance, multiple studies have measured inflammatory mediators such as cytokines and chemokines in schizophrenia patients (10, 11). Additionally, simple inflammatory indices derived from complete blood count data - including the neutrophil-to-lymphocyte ratio (NLR) - have been evaluated as potential biomarkers (5, 12).

Several routine biochemical parameters - including serum iron, hemoglobin, sodium, calcium, glucose, ALT, GGT, and cholesterol levels are currently being investigated as potential biomarkers in schizophrenia patients (13–18). Iron serves as an essential cofactor in the brain’s dopaminergic system, and iron deficiency has been reported to correlate with the severity of negative symptoms in schizophrenia (14). Moreover, anemia is frequently observed in chronic schizophrenia patients, with a more pronounced prevalence among female patients (15). Regarding electrolyte and mineral balance, emerging evidence suggests that disturbances in calcium metabolism may contribute to schizophrenia pathophysiology, with studies consistently reporting lower serum calcium levels in patients (13). Furthermore, alterations in serum sodium levels have also been documented in these patients (16). From a metabolic perspective, schizophrenia patients frequently exhibit elevated glucose levels (17) and demonstrate long-term increases in cholesterol levels attributable to antipsychotic treatment (18). Liver function markers (ALT and GGT levels) frequently show asymptomatic elevations in association with antipsychotic use (19). Collectively, these readily available biochemical parameters show significant potential as adjunctive diagnostic tools in schizophrenia assessment.

Considering the diagnostic challenges and biological heterogeneity of schizophrenia, multimodal approaches combining multiple biomarkers representing distinct pathological mechanisms are believed to provide more reliable results than single-marker strategies. Current evidence suggests that biomarker panels reflecting various pathophysiological processes (e.g., neuroinflammation, metabolic dysfunction, and neurotransmitter abnormalities) could significantly improve clinical practice by enhancing diagnostic accuracy, predicting treatment response, and monitoring disease progression. This integrated multi-biomarker approach is expected to overcome the limitations of current diagnostic methods while providing a more comprehensive biological understanding of the disorder (20, 21). On the other hand, the simultaneous evaluation of multiple biomarkers increases data dimensionality and complexity, pushing the limits of conventional statistical analyses. Identifying hidden patterns in high-dimensional biological data and constructing diagnostic models from them may require substantial computational power. This is precisely where machine learning (ML) techniques become invaluable.

Machine learning offers a more objective and data-driven approach to decision-making by analyzing statistical relationships and patterns within high-dimensional, heterogeneous datasets—capabilities that surpass human intuition. Indeed, machine learning algorithms have been applied in various forms to aid in the diagnosis and prognosis of schizophrenia and related psychiatric disorders (22). A recent comprehensive review further emphasized the effectiveness of AI-based schizophrenia detection methods across multiple modalities including EEG, structural MRI (sMRI), and functional MRI (fMRI). This review highlighted that machine learning approaches such as SVM, Random Forest, and deep learning models (CNN, GAN, CapsNet) have consistently achieved high accuracy (up to 99.5%), underscoring their strong potential for clinical implementation. Nevertheless, the study identified limitations such as the minimal localization of brain regions associated with schizophrenia and recommended future research efforts towards multimodal (EEG and MRI combined) approaches to further enhance diagnostic accuracy (23). Ke et al. developed an integrated machine learning framework combining multi-omics data (gut microbiota, blood biomarkers, and EEG signals) to distinguish schizophrenia patients from healthy controls, achieving 91.7% accuracy and 96.5% AUC using a support vector machine (SVM) algorithm (24). Kozyrev et al. demonstrated that deep neural networks (DNNs) outperformed other machine learning algorithms in terms of both sensitivity and specificity when analyzing peripheral blood biomarkers (including cytokines, chemokines, and growth factors). Their findings further revealed that the combined use of multiple biomarkers significantly enhanced diagnostic efficacy compared to single-marker approaches (5). Fernandes et al. developed a multimodal data integration model incorporating immune, inflammatory, and cognitive biomarkers to differentiate between bipolar disorder and schizophrenia, demonstrating superior performance compared to single-domain approaches (25). In a separate study, Yee et al. successfully developed machine learning models using peripheral inflammatory biomarkers to differentiate between three distinct patient groups: those responding to conventional antipsychotics, those responding specifically to clozapine, and treatment-resistant cases. Their findings demonstrated that SVM algorithms outperformed traditional statistical tests in capturing complex data patterns, while artificial intelligence-based explainability techniques (particularly SHAP analysis) significantly improved model interpretability - a crucial advancement for clinical applications of such predictive models in psychiatric practice (26). Finally, Khoodoruth and colleagues achieved 88.41% accuracy in distinguishing treatment-resistant and non-resistant schizophrenia patients from healthy controls using a random forest algorithm based on routine laboratory inflammatory markers. This study highlights the utility of peripheral biomarkers for early diagnosis and personalized treatment strategies in schizophrenia (27). Similar studies conducted on this topic are summarized in **Table 1**.

**Table 1:** Comparative summary of machine learning studies on schizophrenia diagnosis.

Study	Dataset (Sample Size)	Biomarkers/Features	Methods/Algorithms	Performance Metrics	Biomarker Type
Ke et al. (2021) (24)	SZ: 49, Controls: 50	Microbiota, blood biomarkers, EEG	SVM	Accuracy: 91.7%, AUC: 0.965	Complex, expensive, multi-domain
Kozyrev et al. (2023) (5)	SZ: 217, Controls: 90	Cytokines, chemokines, growth factors	Deep Neural Networks (DNN)	Sensitivity: ~87%, Specificity: ~52%	Complex, laboratory-based immunological
Fernandes et al. (2020) (25)	SZ: 58, BD: 98, Controls: 123	Immune, inflammatory, cognitive tests	Logistic regression, SVM	Sensitivity: 84%, Specificity: 81%	Complex, cognitive & immunological
Yee et al. (2025) (26)	SZ: 146 (with response subtypes), Controls: 49	Peripheral inflammatory proteomics	SVM with SHAP analysis	AUC: 0.74–0.88	Advanced, expensive proteomic panel
Khoodoruth et al. (2024) (27)	SZ (treatment-resistant vs. non-resistant) & Controls	Routine inflammatory laboratory markers	Random Forest	Accuracy: 88.41%	Routine laboratory-based inflammatory

SZ, Schizophrenia; BD, Bipolar Disorder; RF, Random Forest; SVM, Support Vector Machine; KNN, K-Nearest Neighbor; LR, Logistic Regression; DNN, Deep Neural Networks; EEG, Electroencephalography; CBC, Complete Blood Count; SHAP, SHapley Additive exPlanations; GWO, Grey Wolf Optimization; AUC, Area Under the Curve.

The performance of machine learning models largely depends on proper feature selection and effective optimization of model parameters. In this context, nature-inspired optimization algorithms provide efficient and flexible solutions for preprocessing stages such as feature selection, dimensionality reduction, and hyperparameter tuning (28). Developed by Mirjalili et al. in 2014, the Grey Wolf Optimizer (GWO) is a swarm intelligence-based metaheuristic optimization technique. GWO has demonstrated effectiveness in feature selection across diverse data types, including structured clinical data, medical images, and biological signals associated with various diseases (e.g., cardiovascular disorders, diabetes, and cancer) (29–31). This study presents a novel approach to schizophrenia classification by exclusively using routine peripheral blood biomarkers (biochemical and hematological parameters) through an integrated machine learning framework combining GWO with multiple classifiers (Random Forest, SVM, Logistic Regression, and KNN). Unlike previous studies that often rely on specialized, costly, or multi-modal biomarker data, this study uniquely leverages routine clinical blood tests optimized through GWO integrated with various classifiers. The primary aim of this research is to address the critical need for objective, practical, and cost-effective schizophrenia diagnostic tools. By employing GWO for optimal feature selection, we aim to significantly enhance diagnostic performance and clinical interpretability using commonly accessible laboratory parameters. Consequently, our methodology bridges the gap between computational complexity and clinical applicability, providing immediate translational potential and facilitating broader integration into routine psychiatric practice.

## 2 Materials and methods

**Figure 1:**
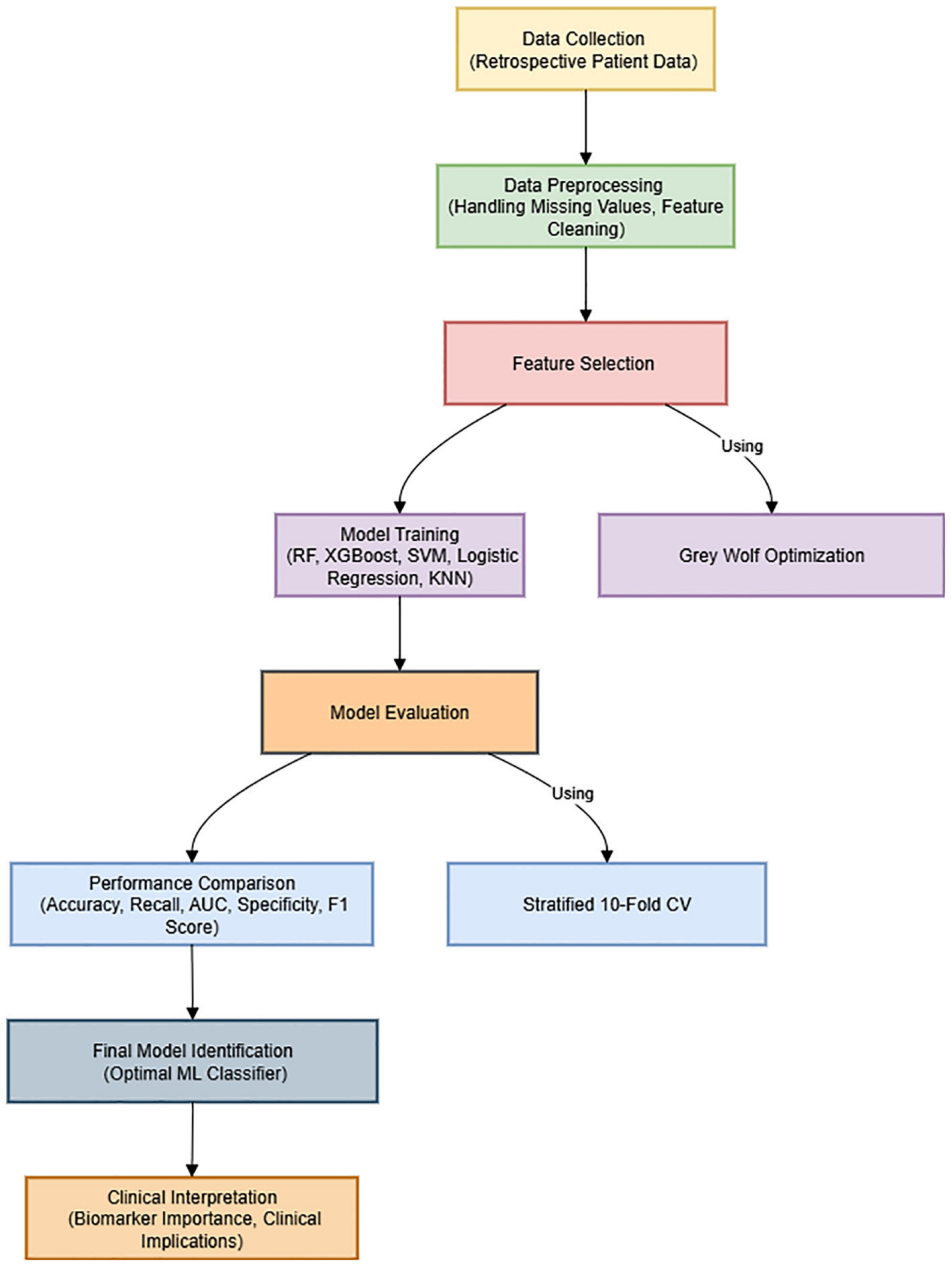
Methodological workflow of the study.

The overall framework of the proposed methodology, including data preprocessing, feature selection using GWO, model training, cross-validation, and performance evaluation, is summarized in **Figure 1**. This visual representation aims to enhance the clarity and readability of the methodological workflow.

### 2.1 Study design and participants

This retrospective case-control study investigated the potential of routine blood parameters to differentiate schizophrenia patients from healthy individuals using archived laboratory and clinical data from the last five years of 203 schizophrenia inpatients at Hitit University Training and Research Hospital and 192 age-matched controls without psychiatric diagnoses selected from hospital records during the same period, forming a total cohort of 395 participants. The study received ethical approval from Hitit University’s Institutional Review Board (Decision No: 2025-54) and was granted exemption from informed consent requirements as it utilized anonymized retrospective data, with all procedures conducted in accordance with ethical guidelines and the Helsinki Declaration.

### 2.2 Laboratory parameters

This study collected comprehensive demographic information (age in years and sex [male/female]) and routine hematological/biochemical parameters from peripheral blood samples for all participants, including complete blood count parameters (white blood cells [WBC], red blood cells [RBC], hemoglobin [HGB], hematocrit [HCT], mean corpuscular volume [MCV], mean corpuscular hemoglobin [MCH], mean corpuscular hemoglobin concentration [MCHC], platelet count [PLT], mean platelet volume [MPV], plateletcrit [PCT], platelet distribution width [PDW], and red cell distribution width [coefficient of variation (RDW-CV) and standard deviation (RDW-SD)]), leukocyte subpopulations and derived ratios (absolute counts [#] and percentages [%] of lymphocytes [LY], neutrophils [NE], monocytes [MO], eosinophils [EO], and basophils [BA], along with immature granulocyte count and percentage [IG# and IG%], nucleated red blood cell count and percentage [NRBC# and NRBC%], and neutrophil-to-lymphocyte ratio [NLR]), and biochemical parameters (fasting glucose, urea, blood urea nitrogen [BUN], creatinine, estimated glomerular filtration rate [eGFR], calcium, sodium, potassium, iron, aspartate aminotransferase [AST], alanine aminotransferase [ALT], gamma-glutamyl transferase [GGT], alkaline phosphatase [ALP], total cholesterol, low-density lipoprotein [LDL] cholesterol, high-density lipoprotein [HDL] cholesterol, and triglycerides), with all laboratory measurements performed on peripheral blood samples collected during hospital admission for schizophrenia patients and during routine health check-ups for controls, analyzed using standard automated laboratory equipment and methods, and extracted from the hospital information system.

### 2.3 Data preprocessing

Before proceeding to the analysis phase, a comprehensive data cleaning and preprocessing process was carried out to make the raw data suitable for modeling. This step plays a critical role in eliminating noise, missing values, and inconsistencies, which directly affect the model’s performance. The process was conducted as follows:

Feature elimination: As part of the preprocessing, missing data were first analyzed; laboratory parameters with more than 50% missing values in the dataset obtained from 395 participants were excluded from the study. This threshold was applied to eliminate variables with low measurement frequency, which could weaken statistical significance and negatively impact the model’s generalization capability.

**Handling missing data:** Missing values in the remaining parameters were imputed to prevent sample loss. For numerical variables, missing values were filled using the median of the available observations for each variable, as the median is robust to outliers and provides a more stable measure in the case of skewed distributions. For the only categorical variable, gender, any missing values were filled using the most frequent category (mode). As a result, complete data were ensured for all 395 records.

**Encoding categorical variables:** The gender variable (female/male) was converted into a binary numerical format using the *LabelEncoder* class to make it suitable for machine learning models.

This structured and systematic preprocessing pipeline cleaned the dataset of inconsistencies and transformed it into a well-organized, standardized format. Consequently, complete data were ensured across all 395 entries, and the model’s predictive power and stability were significantly enhanced.

### 2.4 Feature selection with grey wolf optimization

To reduce data dimensionality and select the most discriminative biomarkers for schizophrenia classification, we employed the GWO algorithm as a wrapper-based feature selection method (32). GWO is a nature-inspired optimization technique that simulates the social hierarchy and cooperative hunting behavior of grey wolves. The algorithm iteratively adjusts candidate solutions (wolves) in the feature space based on guidance from alpha, beta, and delta wolves, corresponding to the top three candidate solutions at each iteration.

While some ensemble-based classifiers, such as Random Forest and XGBoost, inherently perform internal feature selection, we utilized GWO as an external feature selection step prior to model training. This approach aimed to identify a compact, optimal feature subset applicable across various classifiers, including those without robust built-in feature selection capabilities (e.g., Logistic Regression, KNN, and SVM). Thus, external feature selection ensured consistent, interpretable, and comparable feature usage, reduced model complexity, and enhanced clinical applicability.

In this implementation, each wolf represented a candidate subset of features, encoded as a binary vector, where “1” indicated selection, and “0” indicated exclusion of the feature. The quality of each subset (wolf) was evaluated through a fitness function defined as the classification accuracy of a Random Forest classifier, calculated using cross-validation on the training data. The fitness function was formulated as:


$$\text{Fitness }\left(\text{Accuracy}\right)=\frac{\left(TP\;+\;TN\right)\;}{\left(TP\;+\;TN\;+\;FP\;+\;FN\right)}$$

where:

TP: True Positives

TN: True Negatives

FP: False Positives

FN: False Negatives

The GWO algorithm aimed to maximize this accuracy metric while minimizing feature count, thus balancing classification performance and generalizability.

The GWO algorithm was implemented from scratch in Python, ensuring full methodological transparency. Based on preliminary testing for computational efficiency and optimal convergence, we utilized a population size of 10 wolves and executed the algorithm for 20 iterations. During each iteration, wolves updated their feature selections guided by the positions of the alpha, beta, and delta wolves. After completing the iterations, an optimal subset containing 20 features was selected from an initial set of 48 features. This optimal subset, characterized by maximum discriminative power, was subsequently used in model training and evaluation.

**Figure 2:**
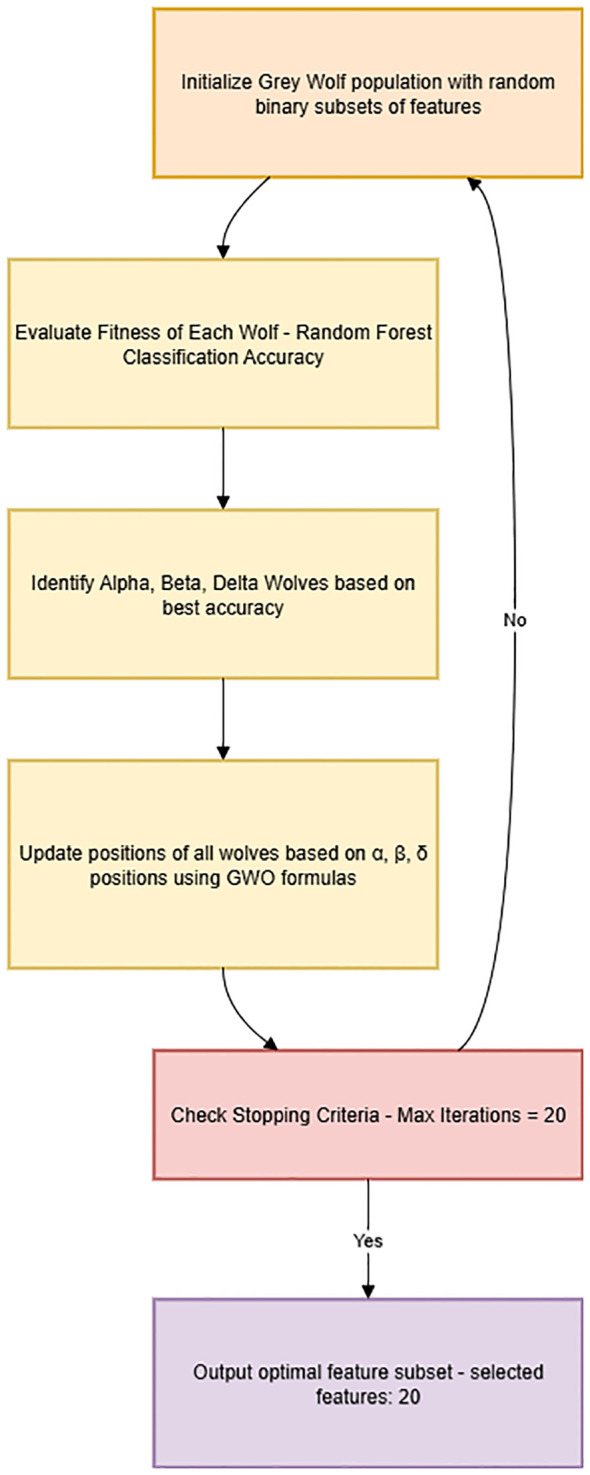
Feature selection process with grey wolf optimization (GWO).

**Figure 3:**
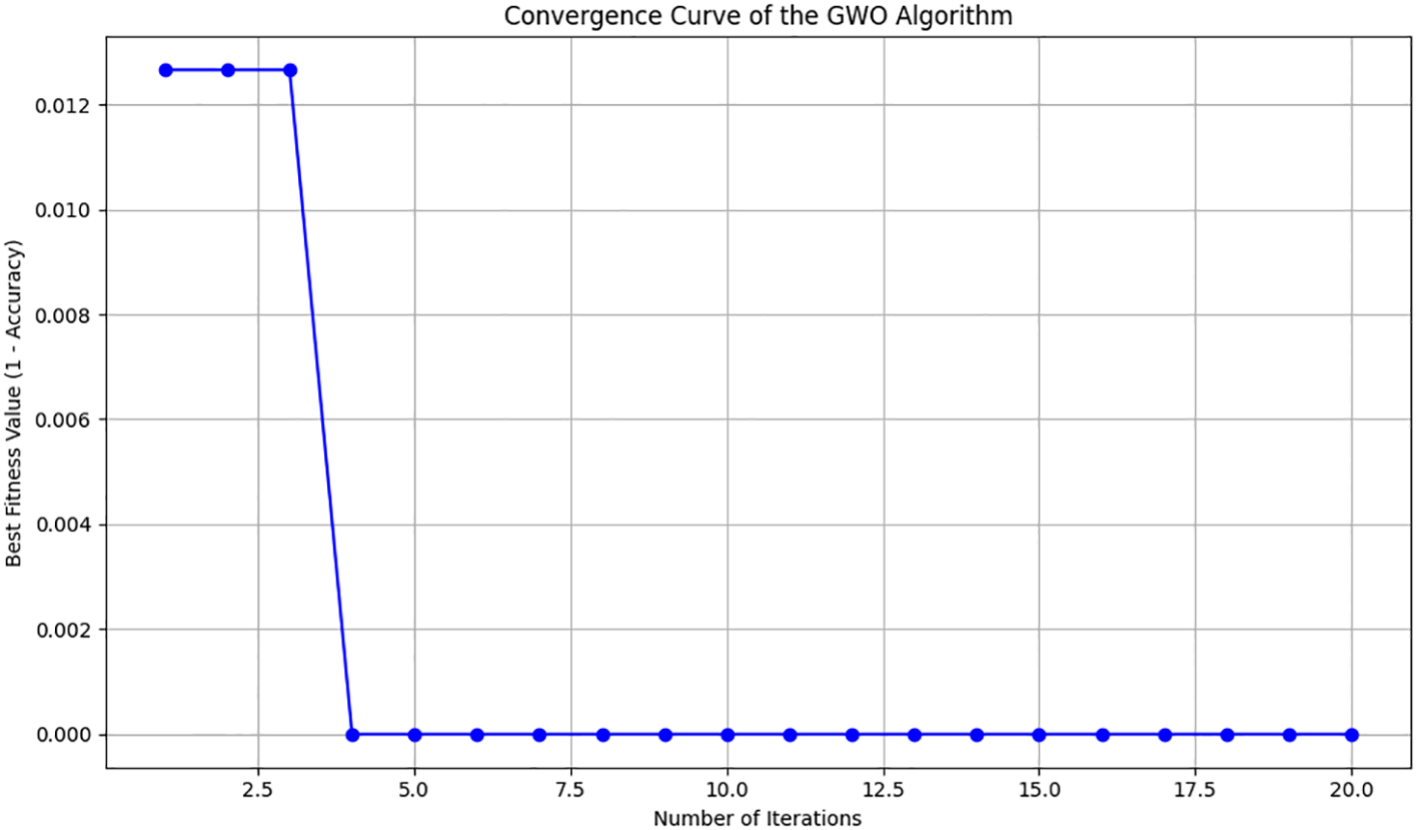
Convergence graph of the GWO algorithm over iterations.

A clear, detailed flowchart explaining the working of the GWO algorithm and feature selection steps was created and included in the manuscript (**Figure 2**), highlighting each step from initialization to final feature subset selection:

For comparative analysis, we evaluated classification performance both with the original full feature set and the GWO-selected feature subset. The overall workflow of GWO-based feature selection is presented in **Figure 3**.

### 2.5 Classification algorithms

Five different classification algorithms were evaluated: RF, LR, SVM, KNN, and XGBoost. These algorithms were chosen based on their common use, robustness, and established performance in prior schizophrenia biomarker studies. All models were implemented in Python using scikit-learn (version 1.6.1) for RF, LR, SVM, and KNN, and the xgboost library for the XGBoost model. The classifiers used were as follows:

**Random Forest (RF):** It is an ensemble learning method composed of multiple decision trees. Each tree is trained using randomly selected samples from the training data, and at each split, it works with a randomly selected subset of features. This ensures independence among the trees and improves generalization performance. Random Forests (RF) are advantageous due to their ability to work well with high-dimensional data and their inherent capability to determine feature importance (33).

Logistic Regression **(LR):** This is a generalized linear model used for binary classification. The probability of belonging to the positive class is modeled using a linear combination of the input features. The model is trained via maximum likelihood estimation of the logistic function. To prevent overfitting, L2 regularization (ridge penalty) is applied by default. The regularization parameter is left at its default value, C = 1.0 (34).

Support Vector Machines (SVM): This is a kernel-based classifier that identifies the optimal separating hyperplane in the given feature space to maximize the margin between two classes. In this study, the Radial Basis Function (RBF) kernel was preferred due to its ability to capture nonlinear relationships. The regularization parameter and kernel parameters for SVM were kept at their default values from the scikit-learn library (C = 1.0, gamma = ‘scale’) (35).

K-Nearest Neighbors (KNN): A non-parametric, instance-based learning method where a sample is classified based on the majority class of its *k* nearest neighbors in the feature space. In this study, *k* was set to 5 (a common default value), and the Euclidean distance metric was used to determine neighbor proximity. Prior to applying KNN, feature scaling (as detailed in Section 2.3) is particularly crucial, as it ensures all features contribute equally to distance calculations (36).

**Extreme Gradient Boosting (XGBoost):** An advanced tree-based ensemble algorithm that builds a collection of decision trees in a sequential process, where each new tree is trained to correct the errors of the previous ones. It employs a regularized objective function (L1/L2 penalties) to prevent overfitting, enhancing generalization performance. Renowned for its computational efficiency and high classification accuracy, XGBoost was implemented using Python’s xgboost library with default hyperparameters (e.g., learning rate, tree depth, number of trees) (37).

All models were implemented in Python using relevant machine learning libraries: scikit-learn was used for RF, LR, SVM, and KNN, while the XGBoost model was implemented using the xgboost library. Each model was first trained with all features, then retrained using the feature subset selected by the GWO algorithm, thereby evaluating the impact of feature selection on classification performance.

### 2.6 Model training and evaluation

The labeled dataset was evaluated using stratified 10-fold cross-validation with 5 repetitions to reliably estimate model performance and preserve class distribution (51% schizophrenia, 49% control). Each classifier was trained on 9 folds and tested on the remaining fold iteratively, with the entire 10-fold process repeated five times to ensure stable and unbiased metrics. Performance metrics reported are the averages of these repetitions. Accuracy is calculated using the following formula:


Accuracy=(TP + TN)/(TP + TN + FP + FN)

In addition to accuracy, other widely-used performance metrics derived from the confusion matrix were analyzed to gain deeper insights into classifier behavior, including:

Sensitivity (Recall): The rate of correctly identifying real schizophrenia patients. The formula:


Sensitivity (Recall)=TP/(TP + FN)

Specificity: It is the rate of correctly identifying real control individuals. The formula:


Specificity=TN/(TN + FP)

Precision: The proportion of true positive predictions among all positive predictions. The formula:


Precision=TP/(TP + FP)

F1-score: It is the harmonic mean of precision and recall, balancing both false positives and false negatives. The formula:


F1 Score=2×(Precision × Recall)/(Precision + Recall)

In the above formulas, TP, TN, FP, and FN are as previously defined in Section 2.4.

Performance metrics including sensitivity, specificity, and accuracy were evaluated to ensure a balanced clinical relevance. Models were compared before and after feature selection using GWO to demonstrate the impact of feature selection on classification accuracy. The analysis was conducted in Python using Pandas for data processing, scikit-learn (version 1.6.1) for model implementation, and the XGBoost Python API. The GWO algorithm was custom-coded, with reproducibility ensured through fixed random seeds. Results were visualized using Matplotlib (version 3.10.1), facilitating rigorous evaluation of routine blood parameters for schizophrenia classification.

## 3 Results

No significant differences were observed between the schizophrenia and control groups in terms of age and sex distribution in the study group (**Table 2**). While the mean age of 203 patients in the schizophrenia group was 28.7 ± 3.9 years, the mean age of 192 participants in the control group was 28.3 ± 10.7 years; this difference in age distributions is not statistically significant (p = 0.6468). While 75.9% of participants in the schizophrenia group were male (154/203), 67.7% were male in the control group (130/192); the difference between these rates was not found to be statistically significant (p = 0.0726). The similarity of the groups in terms of age and gender is a positive result for the homogeneity of the dataset and shows that demographic factors do not create an effect in the comparison of models (**Table 3**).

**Table 2:** Demographic characteristics of schizophrenia and control groups.

Characteristic	HCs (n=192)	SZs (n=203)	p value
Age, mean (SD) (years)	28.3 (10.7)	28.7 (3.9)	0.6468
Sex, Male (%)	130 (67.7%)	154 (75.9%)	0.0726
Sex, Female (%)	62 (32.3%)	49 (24.1%)	

**Table 3:** Comparison of laboratory characteristics of schizophrenia and control groups.

	Control mean	Control Std	Schizoprenia mean	Schizoprenia Std	p
**MO#**	0.53	0.185	0.619	0.237	**0.0001**
**HCT**	44.356	4.207	42.358	4.24	**0.0001**
**HGB**	15.256	1.76	14.371	1.726	**0.0001**
**RBC**	5.112	0.498	4.91	0.471	**0.0001**
**Iron**	53.547	32.252	73.258	40.752	**0.0001**
**Calcium**	9.744	0.182	9.576	0.371	**0.0001**
**Glucose**	93.312	26.932	83.039	10.139	**0.0001**
**Potassium**	4.408	0.171	4.303	0.305	**0.0002**
**MO%**	7.451	1.876	8.153	2.024	**0.0004**
**MCHC**	34.106	1.369	33.602	1.461	**0.0005**
**BA#**	0.016	0.018	0.021	0.017	**0.0009**
**BA%**	0.21	0.225	0.279	0.222	**0.0024**
**GGT**	22.385	6.636	30.212	37.351	**0.0037**
**Triglyceride**	109.932	33.427	142.409	153.837	**0.0037**
**RDW-SD**	40.514	2.654	41.342	2.977	**0.0037**
**IG%**	0.316	0.218	0.389	0.284	**0.0043**
**IG#**	0.025	0.021	0.031	0.024	**0.0048**
**Sodium**	139.807	1.048	140.241	1.969	**0.0062**
**MCH**	29.76	2.202	29.152	2.199	**0.0064**
**RDW-CV**	12.94	1.15	13.215	1.16	**0.0185**
**NRBC#**	0.0	0.001	0.0	0.002	**0.0352**
**NE%**	58.646	8.187	56.64	10.77	**0.0375**
**PLT**	252.594	54.953	241.248	57.168	**0.0453**
**Creatinine**	0.802	0.144	0.83	0.152	0.0855
**NRBC%**	0.001	0.014	0.004	0.022	0.1181
**AST**	22.859	10.654	24.931	15.562	0.1218
**PCT**	0.262	0.049	0.254	0.056	0.1309
**WBC**	7.351	2.72	7.7	2.204	0.1635
**MPV**	10.315	0.805	10.425	0.931	0.2091
**MCV**	86.831	4.376	86.275	4.605	0.2199
**LY%**	31.073	7.887	32.135	9.922	0.239
**ALP**	76.422	20.669	79.271	29.164	0.2614
**EO#**	0.149	0.13	0.163	0.144	0.2863
**NE#**	4.449	1.554	4.601	1.888	0.3807
**LDL Cholesterole**	90.688	12.292	89.084	24.059	0.4011
**LY#**	2.323	1.592	2.428	0.782	0.4117
**tGFH**	118.88	12.021	119.621	9.876	0.5053
**PDW**	12.056	1.668	12.159	2.042	0.5832
**EO%**	2.058	1.744	2.144	1.924	0.641
**HDL Cholesterole**	43.37	4.677	43.665	8.12	0.6561
**ALT**	24.953	19.345	25.778	18.138	0.6625
**Üre**	24.536	6.078	24.281	6.683	0.6908
**Total Cholesterole**	159.719	16.99	160.616	34.498	0.7412
**BUN**	11.401	2.867	11.31	3.154	0.7649
**NLR**	2.082	0.923	2.096	1.351	0.9164

MO#, Monocyte count; HCT, Hematocrit; HGB, Hemoglobin; RBC, Red blood cell; Iron, Serum iron level; Calcium, Serum calcium level; Glucose, Blood glucose level; Potassium, Serum potassium level; MO%, Monocyte percentage; MCHC, Mean corpuscular hemoglobin concentration; BA#, Basophil count; BA%, Basophil percentage; GGT, Gamma-glutamyl transferase; Triglyceride, Serum triglyceride level; RDW-SD, Red cell distribution width-standard deviation; IG%, Immature granulocyte percentage; IG#, Immature granulocyte count; Sodium, Serum sodium level; MCH, Mean corpuscular hemoglobin; RDW-CV, Red cell distribution width-coefficient of variation; NRBC#, Nucleated red blood cell count; NE%, Neutrophil percentage; PLT, Platelet count; Creatinine, Serum creatinine level; NRBC%, Nucleated red blood cell percentage; AST, Aspartate aminotransferase; PCT, Plateletcrit; WBC, White blood cell; MPV, Mean platelet volume; MCV, Mean corpuscular volume; LY%, Lymphocyte percentage; ALP, Alkaline phosphatase; EO#, Eosinophil count; NE#, Neutrophil count; LDL Cholesterol, Low-density lipoprotein cholesterol; LY#, Lymphocyte count; tGFH, Estimated glomerular filtration rate (eGFR); PDW, Platelet distribution width; EO%, Eosinophil percentage; HDL Cholesterol, High-density lipoprotein cholesterol; ALT, Alanine aminotransferase; Üre, Urea; Total Cholesterol, Total serum cholesterol; BUN, Blood urea nitrogen; NLR, Neutrophil-to-lymphocyte ratio.

Laboratory data with the highest significance between control and schizophrenia groups were compared. Mean values and standard deviations are presented, and significant differences between groups are indicated with p-values. Values with p<0.05 were considered statistically significant.

The performance evaluation of machine learning-based models was conducted using 10-fold cross-validation, assessing accuracy, recall (sensitivity), specificity, and F1-score metrics. The application of the GWO algorithm led to minimal changes in the models’ diagnostic performance. Specifically, the Random Forest and XGBoost models maintained consistently high and balanced performance following the implementation of GWO.

Prior to GWO, model accuracy ranged between approximately 65-96%, with XGBoost (95.55%) and Random Forest (94.63%) achieving the highest accuracy values. Before optimization, the XGBoost model demonstrated excellent performance across all metrics: accuracy (95.55%), recall (96.29%), specificity (94.83%), and F1-score (95.72%). Similarly, the Random Forest model showed high accuracy (94.63%), recall (95.00%), specificity (94.29%), and F1-score (94.75%). Logistic Regression (85.44% accuracy) displayed moderate performance, whereas KNN (76.97% accuracy) and particularly SVM (65.52% accuracy) exhibited lower performance.

**Figure 4:**
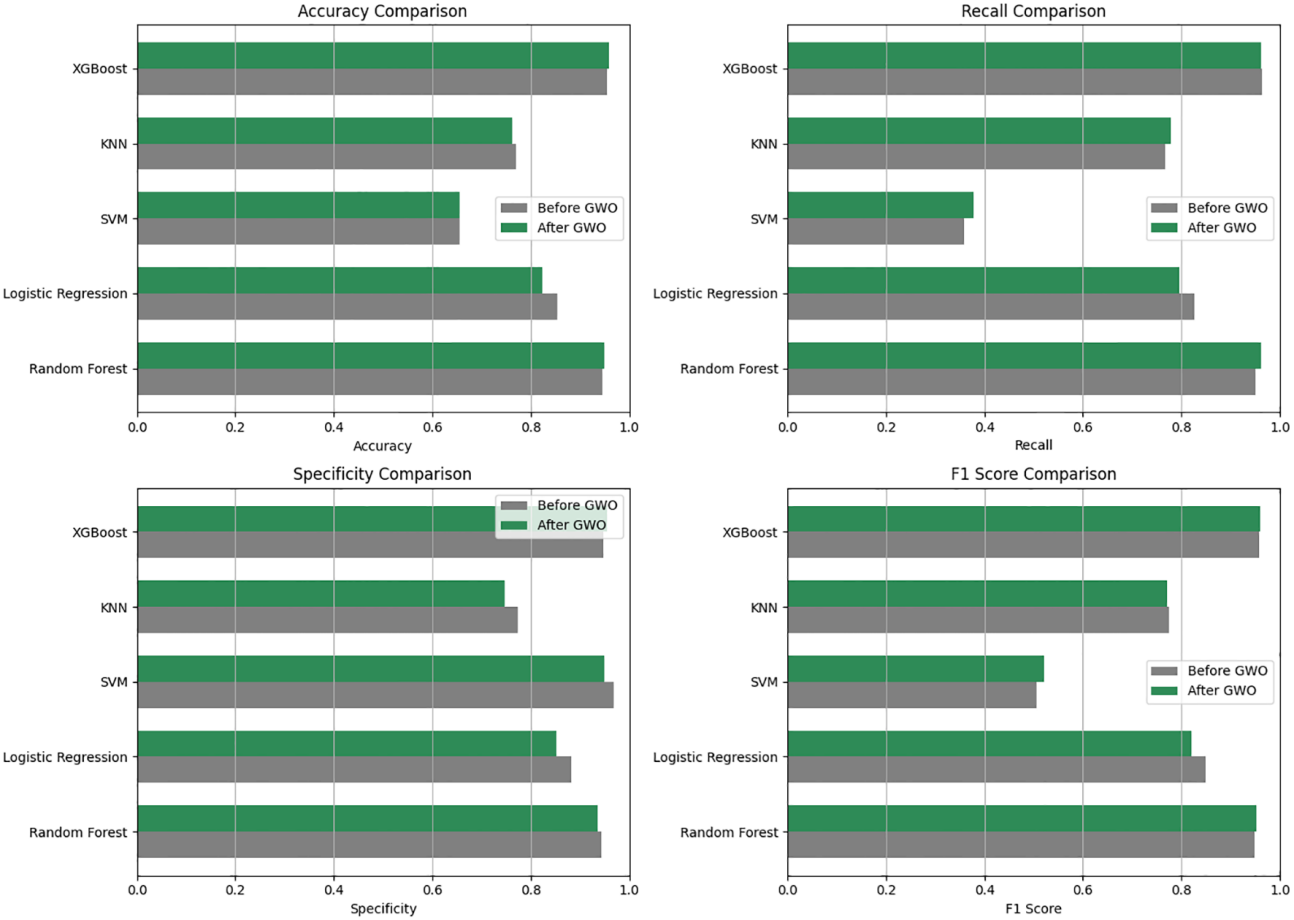
Comparative performances of the machine learning models used before and after GWO optimization in terms of Accuracy, Recall, Specificity and F1 score.

After feature selection with the GWO algorithm, minor variations in model performance were observed (the updated post-GWO ROC curves are shown in **Figure 4**). The Random Forest model slightly improved its accuracy (from 94.63% to 94.95%) and recall (from 95.00% to 96.25%), but specificity decreased slightly (from 94.29% to 93.62%). Similarly, the XGBoost model maintained very high performance, slightly improving accuracy (from 95.55% to 95.90%) and specificity (from 94.83% to 95.54%), while recall remained high (from 96.29% to 96.25%) (**Table 4**).

**Table 4:** Comparative performance results of machine learning models before and after GWO.

Model	Accuracy (Before GWO)	Recall (Before GWO)	Specificity (Before GWO)	F1 Score (Before GWO)	Accuracy (After GWO)	Recall (After GWO)	Specificity (After GWO)	F1 Score (After GWO)
Random Forest	0.9463	0.9500	0.9429	0.9475	0.9495	0.9625	0.9362	0.9514
Logistic Regression	0.8544	0.8265	0.8833	0.8492	0.8226	0.7949	0.8517	0.8197
SVM	0.6552	0.3588	0.9679	0.5051	0.6554	0.3772	0.9492	0.5207
KNN	0.7697	0.7676	0.7746	0.7740	0.7625	0.7787	0.7467	0.7701
XGBoost	0.9555	0.9629	0.9483	0.9572	0.9590	0.9625	0.9554	0.9598

The KNN model showed a minor decline, with accuracy decreasing from 76.97% to 76.25% after GWO. Logistic Regression also experienced a decrease in accuracy (from 85.44% to 82.26%). The SVM model’s performance remained low and practically unchanged, with accuracy stable at around 65.5% (from 65.52% to 65.54%) and recall slightly increasing (from 35.88% to 37.72%). Consequently, the Random Forest and XGBoost models consistently demonstrated superior performance both before and after GWO optimization, significantly outperforming the other evaluated models.

**Figure 5:**
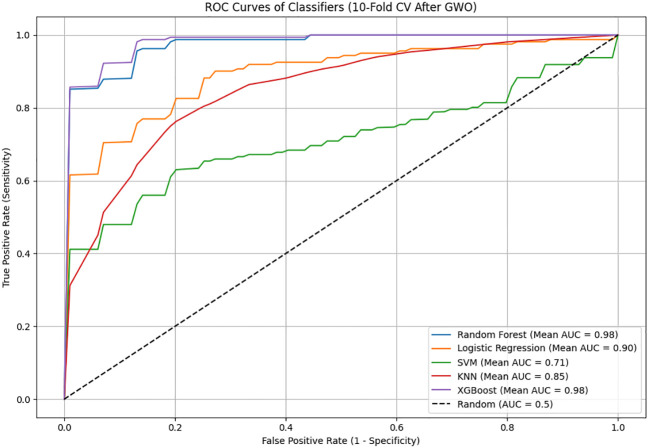
The ROC curves and AUC (Area Under the Curve) values of machine learning models used after Gray Wolf Optimization (GWO).

The performance of machine learning-based models in classifying schizophrenia patients was evaluated using ROC curves and Area Under the Curve (AUC) values, as shown in **Figure 5**. The models were trained using 10-fold cross-validation with features selected after GWO. Among all models, the highest AUC values were achieved by XGBoost (AUC = 0.9866) and Random Forest (AUC = 0.9777), demonstrating excellent diagnostic performance and a very high success rate in distinguishing schizophrenia patients from control individuals. Logistic Regression (AUC = 0.9150) and KNN (AUC = 0.8404) exhibited moderate to high performance, whereas the SVM model (AUC = 0.7756) showed the lowest performance. Examination of the ROC curves indicated that the curves for XGBoost and Random Forest were clearly positioned near the upper-left corner, signifying highly reliable detection of patients with excellent sensitivity and specificity. In conclusion, the exceptionally high AUC values obtained after GWO optimization for the XGBoost and Random Forest models further reinforce their potential as effective and robust diagnostic tools for schizophrenia.

In the Random Forest and XGBoost models, the features contributing the most to the diagnosis of schizophrenia and their importance levels are provided. In the Random Forest model, the most important features were total protein, fasting glucose (FBG), iron, amylase, and creatine kinase (CK), whereas in the XGBoost model, total protein, total bilirubin, creatine kinase (CK), uric acid, and tGFH levels stood out as most significant. This difference indicates that the models capture relationships in the data through different pathways or emphasize different biochemical parameters (**Table 5**).

**Table 5:** Most Important Features in Random Forest and XGBoost Models.

Feature	Importance (Random Forest)	Importance (XGBoost)
T. Protein	0.099114	0.284505
Glukoz (AKŞ)	0.096448	0.043234
Demir	0.059816	0.006948
Amilaz	0.054571	0.041404
Kreatin kinaz (CK)	0.051248	0.113914
tGFH	0.050872	0.058490
ALT	0.050541	0.011205
TDBK	0.036266	0.017997
Total Bilirubin	0.034319	0.128578
RBC	0.033316	0.006103
Sodyum	0.031635	0.045824
Total Kolesterol	0.031537	nan
Kalsiyum	0.029864	0.047750
ALP	0.028073	0.022094
MCH	0.027540	0.009742

## 4 Discussion

This study was conducted to distinguish schizophrenia patients from healthy individuals with high accuracy using low-cost, routine biochemical and hematological blood parameters suitable for clinical use. Blood parameters of schizophrenia patients and the control group were statistically compared, and then the most discriminative features were selected using the GWO algorithm to enhance classification power. The classification performances of RF, SVM, LR, KNN, and XGBoost models trained with these selected feature sets were evaluated comparatively using the 10-fold cross-validation method.

The analysis revealed that the Random Forest algorithm, trained with GWO-selected features, achieved excellent performance with 94.95% accuracy, 96.25% sensitivity, 93.62% specificity, and a notably high AUC of 0.9777. Similarly, the XGBoost algorithm attained even higher performance, achieving 95.90% accuracy, 96.25% sensitivity, 95.54% specificity, and an exceptional AUC of 0.9866. The high sensitivity, specificity, and outstanding AUC scores of both models clearly indicate their potential as highly robust and reliable clinical tools for schizophrenia diagnosis.

On the other hand, Logistic Regression (82.26% accuracy, 0.9150 AUC), KNN (76.25% accuracy, 0.8404 AUC), and especially SVM (65.54% accuracy, 0.7756 AUC) demonstrated lower and more limited performance even after GWO optimization. In conclusion, the results strongly demonstrate that the Random Forest and XGBoost models, enhanced by GWO feature selection, can be effectively and reliably used for schizophrenia diagnosis based solely on routine biochemical and hematological parameters.

In our study, the mean fasting glucose level of the schizophrenia group was unexpectedly found to be lower than that of the control group (≈82 mg/dL vs. 93 mg/dL, p<0.001). However, the literature generally reports that fasting glucose levels may be elevated in schizophrenia patients, particularly in association with antipsychotic treatment ​ (38). In fact, even in first-episode drug-naïve patients, findings of insulin resistance and prediabetes have been reported. Second-generation antipsychotics (particularly clozapine and olanzapine) are well-known to induce hyperglycemia due to their strong appetite-stimulating and weight-gain effects (39). The literature reports that while the prevalence of metabolic syndrome in antipsychotic-naïve schizophrenia patients ranges from 3% to 26%, it rises to significantly higher rates of 32% to 68% in treated patients ​ (39). Therefore, our finding is not entirely consistent with the literature; the lower glucose levels in the schizophrenia group could be attributed to impaired glucose tolerance or stress hyperglycemia in some control subjects, or possibly due to sample limitations.

In the schizophrenia group, the mean triglyceride level was slightly higher compared to the control group, though it was borderline statistically significant (≈154 vs. 124 mg/dL, p≈0.068). No significant differences were observed between groups in terms of total cholesterol, LDL, and HDL levels (p>0.1). The literature indicates that dyslipidemia is a common issue in schizophrenia patients receiving antipsychotic treatment​ (39).

Second-generation antipsychotics in particular may elevate triglyceride and cholesterol levels. The similarity in lipid parameters between our patient group and controls may be attributed to the younger age of patients and potential differences in treatment duration or medication types, which were not accounted for in this study.

Schizophrenia patients showed slightly higher levels of AST (aspartate aminotransferase) (~25 U/L vs 23 U/L, p≈0.12) and ALT (alanine aminotransferase) (~26 U/L vs 25 U/L, p≈0.66) compared to controls. Although the statistical increase in AST was minor, it might indicate subtle differences in liver or muscle cell function. The literature notes that both typical and atypical antipsychotics can cause mild elevations in liver enzymes (19).

Sodium levels were found to be statistically slightly higher in the schizophrenia group (~140.2 mmol/L vs. 139.8 mmol/L, p<0.005). Potassium levels, however, were slightly lower in the schizophrenia group (~4.3 vs. 4.4 mmol/L, p<0.05). Although the between-group differences were statistically significant for both electrolytes, the absolute differences were quite small and do not suggest a clinically meaningful change (values remained within normal reference ranges for both groups).

The higher sodium level was likely an incidental finding, possibly due to hydration status or laboratory measurement variations. The marginally lower potassium in the schizophrenia group might be associated with factors such as dietary habits, potassium intake, or the use of diuretics in some patients.

The results obtained in this study demonstrate performance levels that are comparable to or higher than similar studies utilizing expensive or multiple biological markers. For example, in the study conducted by Ke et al., which evaluated multiple biological markers including microbiota, blood parameters, and EEG data collectively, the best performance was achieved by the SVM algorithm with 91.7% accuracy and a 0.965 AUC value (24). In our study, the XGBoost model trained solely on low-cost routine clinical blood parameters achieved 95.90% accuracy and a 0.9866 AUC value, clearly surpassing the results reported by Ke et al. Similarly, in the multi-domain study by Fernandes et al., which combined blood and cognitive biomarkers, they reported 84% sensitivity and 81% specificity values ​ (25). In our current study, using only simple blood parameters, the Random Forest model achieved 96.25% sensitivity and 93.62% specificity, indicating notably superior performance compared to the results reported by Fernandes et al. Furthermore, the area under the ROC curve (AUC) values in our study were calculated as 0.9866 for XGBoost and 0.9777 for Random Forest, demonstrating comparable or better performance than studies employing significantly more costly and complex biomarkers. This outcome substantially enhances the clinical applicability and practicality of our proposed approach, while offering notable economic advantages.

While our findings support the general consensus in the literature that combining multiple biological data sources enhances diagnostic accuracy, they are particularly significant in demonstrating that remarkably high performance can be achieved using routine clinical blood tests alone (24, 25). This study revealed that tree-based models, particularly Random Forest and XGBoost, achieved high diagnostic performance using an optimal feature subset determined by GWO (AUC = 0.9777 for Random Forest; AUC = 0.9866 for XGBoost), highlighting the direct positive impact of feature selection strategy on model performance. The GWO algorithm effectively scanned the data space to select the most discriminative blood parameters for schizophrenia diagnosis, enabling optimal classification performance even with routine tests. However, small declines were observed in some performance metrics of linear models like Logistic Regression and SVM after GWO application, suggesting that the selected feature subset may not be equally effective for all models. Indeed, while linear models typically benefit from broader feature sets, tree-based models can achieve higher performance with limited but discriminative optimal feature subsets. Consequently, tailoring feature selection methods to the classifier algorithm’s structure is crucial for enhancing diagnostic performance. These findings support that optimization-based methods for selecting biological biomarkers represent a clinically viable and effective approach.

When compared with other studies in the literature, our study offers another significant advantage in terms of clinical applicability and cost-effectiveness. For instance, in the study conducted by Ke et al., specialized and costly tests such as fecal microbiota analysis and EEG were required to achieve high performance (24). Similarly, in the study conducted by Fernandes et al., cognitive tests and blood immunological markers were evaluated together; however, while cognitive tests require expert assessment, specialized laboratory analyses are also needed for immunological markers (25). In the study conducted by Yee et al., SVM models were developed using the Olink^®^ proteome panel to predict antipsychotic treatment response, with reported ROC values ranging between 0.74–0.88 (26)​. The Olink panel represents an advanced technology capable of measuring hundreds of inflammatory proteins, yet it remains a costly method not routinely available in most clinical centers. In contrast, our study achieved comparable or superior performance using standard complete blood count and biochemical parameters routinely measured in clinical practice. This practical advantage offers significant potential for developing a low-cost, widely applicable artificial intelligence tool to support schizophrenia diagnosis.

Since our approach requires no additional sample collection (e.g., stool or brain imaging) and utilizes existing test results, our model could be far more easily integrated into clinical workflows compared to alternative approaches. The methodology’s reliance on routinely available data significantly enhances its real-world applicability while maintaining diagnostic accuracy.

Our study results demonstrate the feasibility of objective biomarker-based approaches for schizophrenia diagnosis. This addresses a well-documented gap in psychiatric practice, where the lack of reliable biological indicators for mental disorders has been consistently emphasized in the literature. Previous efforts have focused on integrating multiple biological data types to develop more reliable diagnostic models (25). Our findings indicate that achieving this objective may be possible without relying on complex and costly multi-modal data. However, certain limitations of our study must be considered. Firstly, due to the retrospective design of our research, certain critical clinical details (detailed medication information (e.g., type, dose, and duration of antipsychotic medication), substance use histories, comorbid medical conditions, and clearly defined inclusion and exclusion criteria) were not consistently available within the archived medical records. The absence of these details could potentially confound biomarker levels and affect the accuracy, reliability, and clinical interpretability of the developed machine learning models. Secondly, our study was limited by the sample size and specific demographic characteristics of the dataset, including a borderline imbalance in sex distribution (p = 0.072), which might influence biomarker levels and model performance. Thirdly, given that disease stages, various antipsychotic treatments, and metabolic conditions may significantly impact routine blood parameters, the robustness of our diagnostic models across these variables remains uncertain. Finally, as our models were validated internally through cross-validation without evaluation on an independent external dataset, concerns related to generalizability and robustness remain. Therefore, external validation with larger, independent, and prospectively designed cohorts incorporating comprehensive clinical and sociodemographic information (including educational background, socioeconomic status, lifestyle factors, and detailed treatment history) is essential to confirm the generalizability and reliability of our findings.

Our study achieved diagnostic accuracy measures comparable to the highest values reported in the literature for schizophrenia diagnosis using a machine learning approach based solely on routine blood tests enhanced by GWO, representing a significant contribution. This methodology demonstrates particular promise for clinical implementation due to its ability to achieve similar performance with substantially simpler data inputs, offering potential to introduce objectivity into diagnostic processes. Specifically, in clinical practice, routine blood test results could be instantly processed through our trained model to provide psychiatrists with supplementary diagnostic support - potentially improving diagnostic accuracy while reducing time-to-diagnosis and enabling earlier intervention. Unlike more complex approaches in the literature, our method’s low cost and high accessibility significantly enhance its potential for widespread healthcare adoption. While the results should be interpreted cautiously given the study’s limitations and generalized prudently, the overall findings strongly suggest that intelligent analysis of routine biochemical and hematological parameters can yield clinically meaningful biomarker panels for schizophrenia, providing a valuable tool suitable for integration into clinical decision-support systems.

## Data Availability

The raw data supporting the conclusions of this article will be made available by the authors, without undue reservation.
